# Optimized spectral filter design enables more accurate estimation of oxygen saturation in spectral imaging

**DOI:** 10.1364/BOE.446975

**Published:** 2022-03-16

**Authors:** Dale J. Waterhouse, Danail Stoyanov

**Affiliations:** 1 Wellcome/EPSRC Centre for Interventional and Surgical Sciences, Department of Medical Physics and Biomedical Engineering, University College London, UK; 2 d.waterhouse@ucl.ac.uk; 3 danail.stoyanov@ucl.ac.uk

## Abstract

Oxygen saturation (SO_2_) in tissue is a crucially important physiological parameter with ubiquitous clinical utility in diagnosis, treatment, and monitoring, as well as widespread use as an invaluable preclinical research tool. Multispectral imaging can be used to visualize SO_2_ non-invasively, non-destructively and without contact in real-time using narrow spectral filter sets, but typically, these spectral filter sets are poorly suited to a specific clinical task, application, or tissue type. In this work, we demonstrate the merit of optimizing spectral filter sets for more accurate estimation of SO_2_. Using tissue modelling and simulated multispectral imaging, we demonstrate filter optimization reduces the root-mean-square-error (RMSE) in estimating SO_2_ by up to 37% compared with evenly spaced filters. Moreover, we demonstrate up to a 79% decrease in RMSE for optimized filter sets compared with filter sets chosen to minimize mutual information. Wider adoption of this approach will result in more effective multispectral imaging systems that can address specific clinical needs and consequently, more widespread adoption of multispectral imaging technologies in disease diagnosis and treatment.

## 1. Introduction

Oxygen saturation (SO_2_) is a crucially important physiological parameter with ubiquitous clinical utility in diagnosis, treatment, and monitoring, as well as widespread use as an invaluable preclinical research tool. Typically, a significant decrease in SO_2_ indicates a disruption of normal biological function. Traditionally, SO_2_ is measured non-invasively using pulse oximetry, or invasively using bedside equipment, such as spectrophotometers, that measure an extracted blood sample. The latter requires a relatively large blood sample, limiting its applicability to measuring arterial or venous blood, and carries inherent drawbacks associated with invasive procedures including risk of infection and use of expensive single-use equipment in addition to workflow challenges arising from the sample acquisition. Neither approach provides spatially resolved information and they are thus unable to resolve local variations in blood saturation that might be useful in assessing certain pathologies. In recent years, research into real-time non-invasive, non-contact optical techniques for visualizing SO_2_ has gained momentum and these techniques have found application in a wide range of indications.

Intraoperative visualization of SO_2_ is valuable across a wide range of surgical specialties. Insufficient perfusion of tissues can result in ischemic injury, reduced viability of repaired tissue and poor healing. Some diseased tissue has a different vascular profile to its healthy form and further, the identification of blood vessels, which may be in unexpected locations due to disease or anatomical variations, is critical in avoiding accidental injuries that might otherwise result in prolonged procedures and serious or life-threatening complications [1]. Moreover, the success of anastomosis, the surgical attachment of two luminal structures [2–4], and the creation of skin flaps [5,6], one of the most common surgical techniques for repairing missing or damaged tissues, rely on ensuring proper tissue perfusion. In the brain, dynamic monitoring of SO_2_ is particularly important to safe and effective cerebrovascular reconstruction [7].

Monitoring SO_2_ is also useful for transplant surgery: for monitoring donor organ perfusion during warm ischemia, whether this be in donor, recipient or during normothermic machine perfusion [8]; and for monitoring reperfusion during surgery [9], ensuring surgical attachment of the organ and its associated vasculature is successful, and promoting long-term viability of the graft.

Because oxygen supply is a fundamental factor for healing, non-invasive visualization of SO_2_ also plays a key role in objective assessment of wound damage and healing potential [10] for heat burns [11,12], chemical burns [13] and radiation burns [14], as well as for diagnosis and monitoring of chronic wounds [15], such as chronic skin ulcers [16] and diabetic foot ulcers [17,18], and for monitoring hemodynamic disorders such as scleroderma and Dupuytren’s contracture [19].

Imaging of SO_2_ is particularly suited to cancer imaging as angiogenesis, the development of tumor-associated neovasculature, is one of the hallmarks of disease [20]. Mapping of SO_2_ might exploit these changes for early detection of cancer, defining lesions based on changes in tissue oxygenation or vascular properties [21]. It can also be used to characterize hypoxia in solid tumors, which is related to treatment-resistance and selection of appropriate therapeutic strategies [22]. Similarly, it has also been used to aid diagnosis and monitoring of pathologic conditions of the retina and optic nerve, where loss of normal oxygen supply is believed to play an important role in disease [23,24].

Beyond application in the clinical setting, visualization of SO_2_ is useful in basic research, for example, mapping of hemodynamic response in the brain to understand brain organization and processing [25] or response to blast-induced traumatic brain injury [26], and for assessment and monitoring in tissue engineering.

Hyperspectral imaging [27–29] (HSI) has the potential to non-invasively measure SO_2_ based on the distinct absorption and scattering spectra of oxyhemoglobin (HbO_2_) and deoxyhemoglobin (Hb). HSI captures both spatial (x,y) and spectral (wavelength,λ) information to acquire an image (hyper)cube (x,y,λ). Spectral unmixing is then used to estimate the abundances of HbO_2_ and Hb in each image pixel based on the captured spectra. By measuring their relative abundance, SO_2_ can be estimated, while the sum of abundances indicates the total blood volume. This approach benefits from being real-time, non-invasive, non-contact and label-free.

Four main acquisition approaches are used: point scanning, where complete wavelength information is captured from each image point sequentially, line-scanning, where complete wavelength information is acquired from each image line sequentially, wavelength scanning, where an entire image is captured at each wavelength sequentially, and snapshot approaches, where the entire image cube is captured in a single snapshot. With all approaches, there is a trade-off between speed, spatial resolution and spectral resolution. Devices with high spectral and spatial resolution tend to be slow, bulky, costly and susceptible to misalignment – unsuitable for a clinical environment where low-cost, robust, real-time imaging is desirable [30]. Additionally, capturing more wavelengths at higher resolution leads to larger image cubes, requiring larger storage systems, longer save times, and slower classification and display. For these reasons, techniques intended for clinical translation tend to capture a reduced number of narrow wavelength ‘bands’ and thus perform ‘multispectral imaging’ (though there is no universally agreed limit on the number of bands required to distinguish hyper/multi-spectral).

Multispectral imaging is typically implemented by wavelength scanning, either using a fast filter wheel or tunable light source, or more recently by using spectrally resolved detector arrays (SRDAs), exploiting spectral filters deposited directly onto the imaging detector in a mosaic pattern. Whichever acquisition regime is used, a fundamental question remains: which wavelengths should be captured to visualize SO_2_ with maximum accuracy?

Typically, this question has been avoided in the development of biomedical multispectral imaging, with developers deploying general purpose ‘off-the-shelf’ sensors with ∼10 evenly spaced narrow bands across the wavelength range of interest [27–29]. One of the key challenges is the lack of reliable gold-standard datasets of biological tissue spectra [30], so those developers that have attempted to address the question of spectral band selection have done so using a 2-stage development process, first capturing high resolution data from their tissue of interest using slow scanning HSI devices, before using the high-resolution data to perform spectral band selection for MSI.

This approach has seen some promising successes. Wirkert et al. (2014) analyzed surgical image cubes comprised of 30 wavelengths and used an information-theory-based approach to identify seven optimal bands for SO_2_ estimation, allowing them to perform MSI with a fast filter-wheel device [31]. Waterhouse et al. (2021) analyzed diffuse reflectance spectra to identify three bands that would optimally display contrast between Barrett’s esophagus and cancer in the esophagus, achieving >12-fold contrast enhancement [21]. Perhaps the biggest success of the 2-stage approach, and certainly the most clinically used in routine care, is demonstrated by the invention of narrow band imaging (NBI). The 2 filters used for NBI were selected from 9 off-the-shelf filters for their ability to enhance contrast for vasculature in the human tongue [32]. Even with this relatively crude selection of only two bands, NBI has proven advantageous in the detection and characterization of early Barrett’s esophagus–related neoplasia. It was the first advanced imaging technique to meet the requirements for recommendation in Barrett’s esophagus surveillance [33] and has been successfully translated into routine clinical practice.

From a statistical point of view, spectral band selection is viewed as an optimization problem in which we seek a subset of spectral bands that capture most of the information for a particular hyperspectral signal, whilst removing noise and spectral redundancy (highly correlated wavelengths). However, for clinical application, it may not be necessary to collect *all* the information, as much of it may be non-discriminatory. In this sense, spectral band optimization for clinical application aims to eliminate noise and spectral redundancy whilst preserving discriminant (or diagnostic) information only.

Spectral band selection has been developed for decades in the signal processing community [34], particularly for application in land surveillance, but few of the techniques that have been developed in this field have been applied to biomedical imaging problems. Where spectral band optimization has been applied to biomedical problems, researchers aimed to maximize accurate classification of tissues. However, rather than directly maximize classification performance itself, which is computationally expensive as it requires classifiers be trained and tested for each filter set, the studies used indirect ‘functions of merit’. Some studies maximized mutual information [31,35,36], which neglects the above-mentioned subtlety that not *all* information is *useful* information; others maximized differences between the characteristic spectra of classes, such as root-mean-square-difference or variance [37], but this is not applicable to continuous gradient problems such as estimating SO_2_. Borrowing vocabulary from feature selection for machine learning, both methods are ‘filter’ methods rather than ‘wrapper’ methods, meaning that whilst each selected band contributes to maximizing the chosen function of merit, there is no guarantee that selected filters will result in the best performance once applied to the final classification problem.

In this paper, we compare ‘off-the-shelf’ filter sets to those selected using optimization approaches; both indirect optimization by maximization of root-mean-square-difference, spectral angle and mutual information, and direct optimization by minimization of error in estimated abundance. Through these comparisons, we demonstrate the merits of direct application-specific spectral band selection and advocate for future work in this area to push multispectral imaging to reach its full potential to impact clinical care through imaging systems tailored to the clinical target and needs.

## 2. Methods

### 2.1 Framework for optimizing spectral filter sets

**Fig. 1.**
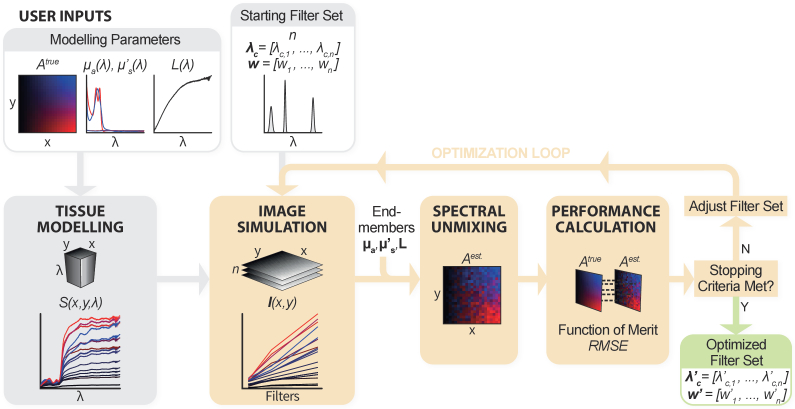
**Overview of the spectral filter optimization framework.** A tissue signal hypercube, *S(x,y,λ)*, is modelled using a ground truth abundance map, *A^true^*, to determine the abundance of oxy- and deoxy- hemoglobin in each pixel, and an empirical model to calculate the spectrum of diffusely reflected light in each pixel using the absorption and scattering coefficients of oxy- and deoxy- hemoglobin, *μ_a_* and *μ’_s_*, and the spectrum of the light source, *L(λ).* Multispectral images, ***I*** *(x,y),* are simulated by propagating the tissue hypercube through *n* filters defined by center wavelengths *λ_c_ = [λ_c,1_, …, λ_c,n_]* and full width half maxima *w = [w_1_, …, w_n_]*. Pixel-wise spectral unmixing of the simulated images results in an estimated abundance map, *A^est.^(x,y)* and this is compared to the ground truth abundance map to determine the root-mean-square-error (*RMSE*). The *RMSE* is minimized by adjusting the filter parameters in an optimization loop until predefined stopping criteria are met, resulting in an optimized filter set.

An overview of our approach to optimizing spectral filter sets is shown in Fig. 1. Briefly, a tissue signal hypercube, *S(x,y,λ)*, is modelled using a ground truth abundance map, *A^true^*, to determine the abundance of oxy- and deoxy- hemoglobin in each pixel, and an empirical model to calculate the spectrum of diffusely reflected light in each pixel using the absorption and scattering coefficients of oxy- and deoxy- hemoglobin, *μ_a_* and *μ’_s_*, and the spectrum of the light source, *L(λ).* Multispectral images, ***I*** *(x,y),* are simulated by propagating the tissue hypercube through *n* filters defined by center wavelengths *λ_c_ = [λ_c,1_, …, λ_c,n_]* and full width half maxima *w = [w_1_, …, w_n_]*. Pixel-wise spectral unmixing of the simulated images results in an estimated abundance map, *A^est.^(x,y)* which is compared to the ground truth abundance map to determine the root-mean-square-error (*RMSE*). The *RMSE* is minimized by adjusting filter parameters in an optimization loop until predefined stopping criteria are met, resulting in an optimized filter set.

### 2.2 Tissue modelling to generate signal hypercubes

Signal hypercubes were simulated as follows. A ground truth abundance map, *A^true^(x,y,i)*, is defined such that,

(1) $$A^{true}(x,y,1)=A_{HbO_{2}}^{true}(x,y)$$

(2) $$A^{true}(x,y,2)=A_{Hb}^{true}(x,y)$$

where *x* and *y* are spatial coordinates in the map, $A_{HbO_{2}}^{\text{true}}\;(x,y)$ are the ground truth abundances of oxyhemoglobin (HbO_2_) and deoxyhemoglobin (Hb) at the point *(x,y)* respectively. This map had a size of 21 × 21 pixels. The oxygen saturation, $SO_{2}^{\text{true}}\;(x,y)$ , at the point *(x,y)* is determined as,

(3) $$SO_{2}^{true}(x,y)=\frac{A_{HbO_{2}}^{true}(x,y)}{A_{HbO_{2}}^{true}(x,y)+A_{Hb}^{true}(x,y)}$$

and the total abundance of hemoglobin (THb) is defined as,

(4) $$A_{THb}^{true}(x,y)=A_{HbO_{2}}^{true}(x,y)+A_{Hb}^{true}(x,y)$$

The map was defined such that a linear gradient of SO_2_ (from 0 to 1) was present horizontally across the map, while a linear gradient of THb (from 0 to 1) was present vertically across the map, thus ensuring all combinations of SO_2_ and THb are evenly represented in the map.

To simulate the signal detected from diffuse reflectance imaging of the ground truth abundance map, we considered several methods of calculating tissue reflectance from absorption and scattering coefficients. These included calculation from the effective attenuation coefficient [38], a model of diffuse reflectance under diffuse illumination using three flux theory [38], a model of diffuse reflectance using a broad beam model [38] and an empirical model [39]. By comparing the modelled reflectance to real tissue spectra [21], we determined the empirical model to be the best approximation for diffuse reflectance imaging of *in vivo* biological tissue (Fig. S1).

For each pixel in the abundance map, the empirical model is used to calculate the signal for each pixel of a signal hypercube,

(5) $$S(x,y,\lambda )=L(\lambda )\frac{{\mu^{\prime }}_{s}(x,y,\lambda )}{k_{1}\cdot \mu_{a}(x,y,\lambda )+k_{2}}$$

where,

(6) $$\mu_{s}^{\prime }(x,y,\lambda )=A_{HbO_{2}}^{true}(x,y)\mu_{s,HbO_{2}}^{\prime }(\lambda )+A_{Hb}^{true}(x,y)\mu_{s,Hb}^{\prime }(\lambda )$$

(7) $$\mu_{a}(x,y,\lambda )=A_{HbO_{2}}^{true}(x,y)\mu_{a,HbO_{2}}(\lambda )+A_{Hb}^{true}(x,y)\mu_{a,Hb}(\lambda )$$

where *L(λ)* [unitless] is the illumination spectrum, *k_1_* [unitless] and *k_2_* [cm^-1^] are constants, *μ’_s, HbO2_* [cm^-1^] and *μ’_s, Hb_* [cm^-1^] are the reduced scattering coefficients of HbO_2_ and Hb respectively, and *μ_a, HbO2_* [cm^-1^] and *μ_a, Hb_* [cm^-1^] are the absorption coefficients of HbO_2_ and Hb respectively. The illumination spectrum was taken from literature values for a broad band halogen light source as these are commonly used for broadband illumination in spectral imaging. The reduced scattering and absorption coefficients of HbO_2_ and Hb were taken from literature values [40]. The values of *k_1 _*= 0.26 and *k_2 _*= 14 were determined by fitting the empirical model to the mean of 320 spectra captured from *in vivo* human esophageal tissue in a previous study [21] (Fig. S1). Gaussian white noise was added to each spectrum using the MATLAB function ‘awgn’ with a signal to noise ratio of 100. This results in a hypercube of spectral data, *S(x,y,λ)*.

### 2.3 Simulation of multispectral imaging

For simulated imaging, a set of *i = 1, …, n* spectral filters are defined as,

(8) $$F=\left[\begin{array}{ccc} F_{1}(\lambda ) & \cdots & F_{n}(\lambda ) \end{array}\right]$$

where,

(9) $$F_{i}(\lambda )=N\;\text{exp}\left[-4\;\ln(2)\;{\left(\frac{\lambda -\lambda_{c,i}}{w_{i}}\right)}^{2}\right]$$

where *N* is a normalization factor that ensures the area under the curve is equal to 1, *λ_c,i_* is the center wavelength of the *i*th filter, and *w_i_* is the full width half maximum (FWHM) of the *i*th filter such that,

(10) $$\lambda_{c}=\left[\begin{array}{ccc} \lambda_{c,1} & \cdots & \lambda_{c,n} \end{array}\right]$$

(11) $$w=\left[\begin{array}{ccc} w_{1} & \cdots & w_{n} \end{array}\right]$$

are the center wavelengths and FWHMs of the filter set ***F***.

To simulate imaging with the filter set, the signal hypercube, *S(x,y,λ)*, an image cube is calculated,

(12) $$I(x,y)=\left[\begin{array}{ccc} I_{1}(x,y) & \cdots & I_{n}(x,y) \end{array}\right]$$

where,

(13) $$I_{i}(x,y)=\int_{-\infty }^{+\infty }S(x,y,\lambda )\;F_{i}(\lambda )\;\text{d}\lambda$$

Zero-mean gaussian white noise was added to each image using the MATLAB function ‘imnoise’ with variance 5 × 10^−5^. To simulate the auto exposure function present in most imaging systems, the image cube was normalized to the maximum pixel value in the whole image cube.

### 2.4 Spectral unmixing to estimate oxygen saturation

For spectral unmixing, the reduced scattering coefficients, the absorption coefficients and the illumination spectrum are first propagated through the spectral filters to generate endmembers,

(14) $${\mu^{\prime }}_{s,HbO_{2}}=\int_{-\infty }^{+\infty }\mu_{s,HbO_{2}}^{\prime }(\lambda )\;F(\lambda )\;\text{d}\lambda$$

(15) $${\mu^{\prime }}_{s,Hb}=\int_{-\infty }^{+\infty }\mu_{s,Hb}^{\prime }(\lambda )\;F(\lambda )\;\text{d}\lambda$$

(16) $$\mu_{a,HbO_{2}}=\int_{-\infty }^{+\infty }\mu_{a,HbO_{2}}(\lambda )\;F(\lambda )\;\text{d}\lambda$$

(17) $$\mu_{a,Hb}=\int_{-\infty }^{+\infty }\mu_{a,Hb}(\lambda )F(\lambda )\;\text{d}\lambda$$

(18) $$L=\int_{-\infty }^{+\infty }L(\lambda )\;F(\lambda )\;\text{d}\lambda$$

While the ground truth absorption and scattering spectra might not be known for some applications, it is reasonable to suggest these could be measured in a calibration step, or otherwise calculated using databook values.

For each pixel of the image cube, ***I*** *(x,y)*, the estimated abundances of HbO_2_ and Hb, $A_{HbO_{2}}^{est.}(x,y)$ and $A_{Hb}^{est.}(x,y)$ , are estimated by least squares fitting of the image cube spectra with the empirical model described in Eq. (5–7), using the endmember spectra described in Eq. (14–18) as inputs. In other words, by minimization of the sum of square errors cost function,

(19) $$SSECF(x,y)={\left|I(x,y)-cL\frac{A_{HbO_{2}}^{est.}(x,y){\mu^{\prime }}_{s,HbO_{2}}+A_{Hb}^{est.}(x,y){\mu^{\prime }}_{s,Hb}}{k_{1}[A_{HbO_{2}}^{est.}(x,y)\mu_{a,HbO_{2}}+A_{Hb}^{est.}(x,y)\mu_{a,Hb}]+k_{2}}\right|}^{2}$$

the abundances $A_{HbO_{2}}^{est.}(x,y)$ and $A_{Hb}^{est.}(x,y)$ , can be estimated. The fitted scalar *c* accounts for normalization of the image cube. The fitted abundances form the estimated abundance map, $A^{est}\cdot (x,y,i)$ , with,

(20) $$A^{est.}(x,y,1)=A_{HbO_{2}}^{est.}(x,y)$$

(21) $$A^{est.}(x,y,2)=A_{Hb}^{est.}(x,y)$$

### 2.5 Merit function for performance calculation

To quantify the performance of a particular filter set, the root-mean-square-difference between the estimated abundances and the ground truth abundances was calculated as,

(22) $$RMSE=\sqrt{\frac{1}{2XY}\sum_{x=1}^{X}\sum_{y=1}^{Y}\sum_{i=1}^{2}{\left[A^{est.}(x,y,i)-A^{true}(x,y,i)\right]}^{2}}$$

where *X* and Y are the length and width of the image in number of pixels.

### 2.6 Optimization

To find the optimum filter set the merit function [Eq. (22)] is minimized. We consider the selection of *n* filters from a discrete set of filters with center wavelengths and FWHMs,

(23) $$\lambda_{c}=470,\;475,\;480,\;\ldots ,\;850\;\text{nm}$$

(24) w=5,10,15,20nm

giving a total of 77 × 4 = 308 possible filters. The problem is then a discretized minimization problem with 2*n* input variables. As the number of filters, *n*, increases, the size of the minimization space (the number possible filter sets) increases rapidly. Ignoring the removal of overlapping bands, there are

(25) $$\frac{77!}{n!\;(77-n)!}\cdot 4^{n}$$

possible filter sets. For *n *= 3, this is 4.7 × 10^6^; for *n *= 4, 3.5 × 10^8^; and for *n *= 5, 2.0 × 10^10^. Thus, an exhaustive approach to optimizing the filter set is not feasible.

#### 2.6.1 Gradient descent

A gradient descent algorithm was used to minimize the merit function and thus find the optimum filter set. Gradient descent algorithms start from an initial estimate for the optimum parameters, in this case the filter properties ***λ_c_*** and ***w***, typically obtained using a linear solver. From here, the algorithm takes repeated steps down the steepest local gradient in the merit function, with the aim of reaching a global minimum. The algorithm started with *n* filters with evenly spaced center wavelengths and *w* = 5 nm.

The gradient descent algorithm moves each filter, *i*, sequentially. Briefly, the merit function [Eq. (22)] is calculated in a small region around the current filter position defined by,

(26) $$\lambda_{c,i}\to \lambda_{c,i}+[-10,\;-5,\;0,\;+5,\;+10]\;\text{nm}$$

(27) $$w_{i}\to w_{i}+[-10,\;-5,\;0,\;+5,\;+10]\;\text{nm}$$

The filter is moved to the position in this region where the merit function is minimized. The occurs sequentially for each filter, *i*, until convergence (defined as no change in merit function for all filters, or a loop returning to a previously tested filter set). Throughout this process, overlapping filters are not allowed,

(28) $$|\lambda_{c,i}-\lambda_{c,j}|\geq \frac{1}{2}(w_{i}+w_{j}),\;\;\;i\neq j$$

#### 2.6.2 Genetic algorithm

**Fig. 2.**
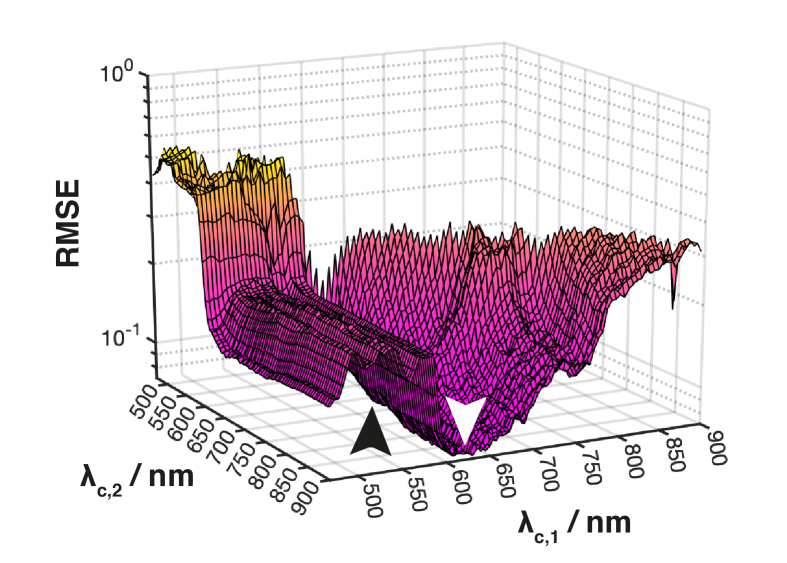
**The complete optimization space for *n* = 2 filters.** The space is very non-smooth with many local minima. The global minimum is at *λ_c,1_* = 625 nm and *λ_c,2_* = 880 nm (white arrow). Gradient descent finds a local minimum at *λ_c,1_* = 610 nm and *λ_c,2_* = 745 nm (black arrow). The genetic algorithm finds the global minimum. All for FWHM = 5 nm.

Gradient descent algorithms are susceptible to getting stuck in local minima and thus not finding a global minimum (Fig. 2). To overcome this limitation, a genetic algorithm was used for optimization. Inspired by biological evolution by natural selection, genetic algorithms start with an initial population of individuals, in this case a population of 20*n* (up to max 100) filter sets with filter parameters ***λ_c_*** and ***w*** generated uniformly at random within the bounds. For these, the ‘fitness’ is calculated using the merit function described in Eq. (22). Subsequent populations are generated based on the current population through three processes: mutation, introducing random changes in properties; crossover, combining the properties of pairs of parents; and automatic unchanged survival of the ‘elite’ members of the population. The algorithm terminates when the average change in the fitness value is below a set tolerance, indicating convergence. Overlap of bands was prevented by passing the inequality in Eq. (28) to the optimization function, effectively preventing such filter sets from being allowed in the population. The crossover fraction was set to 20% for the first 25 generations to search primarily at random via mutation, thus searching the entire optimization space, and 80% thereafter to search primarily via combination of the remaining members of the population.

### 2.7 Alternative merit functions based on maximizing the difference between HbO_2_ and Hb

Previous attempts to optimize spectral filter sets have focused on maximizing the difference between the endmember spectra, such as root-mean-square-difference. These methods presume that maximizing the difference between the extremes of oxygenation (100% and 0%) will lead to greater performance in unmixing the signal contributions of HbO_2_ and Hb, thus leading to more accurate estimation of SO_2_, but it is not clear if this is the case. Thus, to compare our approach of directly optimizing RMSE in SO_2_ [Eq. (22)] to indirect methods, optimization was performed using two alternative performance metrics.

Images were simulated as described in Sections 2.1–2.4, but with the SO_2_-gradient ground truth abundance map replaced with a binary ground truth abundance map consisting of one half having SO_2 _= 1 and the other half having SO_2 _= 0. This allowed the calculation of HbO_2_ and Hb endmember spectra, ***HbO_2_*** and ***Hb***, the mean endmember spectra in the SO_2_ = 1 and SO_2 _= 0 regions respectively. Optimization was performed as described in Section 2.6.2 using a genetic algorithm, with the performance metric in Section 2.5 [Eq. (22)] replaced by two alternative metrics: a metric based on the root-mean-square-difference (RMSD) between HbO_2_ and Hb endmember spectra,

(29) $$RMSD=1-\sqrt{\frac{1}{9}{\left\|\frac{HbO_{2}}{|HbO_{2}|}-\frac{Hb}{|Hb|}\right\|}^{2}}$$

and a metric based on the spectral angle (SA) between HbO_2_ and Hb endmember spectra,

(30) $$SA=\frac{HbO_{2}\cdot Hb}{\|HbO_{2}\|\|Hb\|}$$

where || || represents the Euclidean norm and | | represents the 1-norm.

### 2.8 Optimizing filter sets using mutual information

An alternative approach to selecting filters is to minimize the mutual information measured by the chosen filters. Briefly, for the signal hypercube, *S*, generated according to Eq. (5) with *λ* = [470, 485, …, 850] nm, a distance measure based on normalized mutual information [41] was defined as,

(31) $$D_{i,j}={\left[1-\sqrt{NMI(S(\lambda_{i}),\;S(\lambda_{j}))}\right]}^{2}$$

with the normalized mutual information defined as,

(32) $$NMI(S(\lambda_{i}),\;S(\lambda_{j}))=2\frac{H(S(\lambda_{i}))+H(S(\lambda_{j}))-H(S(\lambda_{i}),S(\lambda_{j}))}{H(S(\lambda_{i}))+H(S(\lambda_{j}))}$$

where *S(λ_i_)* and *S(λ_j_)* are images in the signal hypercube at two different wavelengths *λ_i_* and *λ_j_*, *H(S)* are the marginal entropies of the images; and *H(S_i_, S_j_)* is the joint entropy of the images *S(λ_i_)* and *S(λ_j_)*. Entropies were calculated using the histogram method [41] with 100 bins.

Based on this distance measure, agglomerative hierarchical clustering of the filter images was performed to generate *n* clusters, *C*. The resulting clusters represent mutually exclusive clusters of highly correlated filter images. To calculate distances between pairs of clusters, the average distance between all pairs of images in the two clusters was used.

Finally, the weight of each filter image within a cluster *C* is defined as,

(33) $$W_{i}=\frac{1}{n_{c}}\cdot \sum_{j\in C,\;j\neq i}\left(\frac{1}{\varepsilon +{D_{i,j}}^{2}}\right)$$

where *n_c_* is the number of filter images within the cluster and ɛ = 10^−6^ to avoid singular values. The filter corresponding to the filter image with the highest weight in the cluster is defined as the selected filter from the cluster. Thus, from *n* clusters, *n* filters are selected.

### 2.9 Assessment of optimized filter sets using test hypercubes

**Fig. 3.**
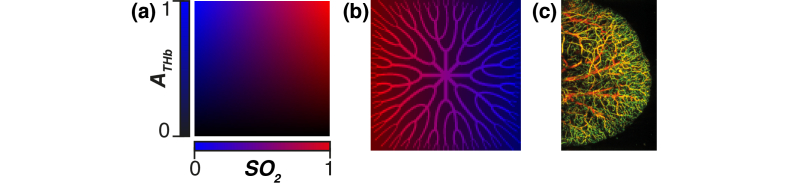
**The ground truth abundance maps of simulated test hypercubes. (a)**. The gradient abundance map used to optimize the filter sets. For optimization this had a size 21 × 21. For testing, this had a size 101 × 101 pixels. **(b).** The radial vascular abundance map used to enable qualitative assessment of performance in a biologically inspired image. This has size 100 × 100 pixels. **(c).** An example image of tissue vasculature that inspired the radial vascular abundance map in B (courtesy of Lina Hacker, University of Cambridge).

To compare filter sets, a series of simulated test hypercubes were prepared using ground truth maps according to Eq. (5–7) (Fig. 3).

For biomedical applications, accurate visualization of oxygen saturation is crucial to enabling the detection of ischemia. To quantify the accuracy of each filter set in determining the oxygenation at different total hemoglobin abundances, a gradient map with a linear gradient of oxygen saturation, $SO_{2}^{\text{true}}$ , (from 0 to 1) horizontally across the map, and a linear gradient of total hemoglobin abundance, $A_{THb}^{\text{true}}$ , (from 0 to 1) vertically across the map, was used. For testing, this map had a size of 101 × 101 pixels. This ensures all combinations of oxygen saturation and total hemoglobin abundance are evenly represented in the map (note, this is a larger version of the phantom used in optimization, which was 21 × 21 pixels) [Fig. 3(a)]. Test images with various degrees of gaussian white noise added to the signal hypercube and various degrees of gaussian white noise added to the simulated images were also generated to assess the effect of noise on filter set performance.

For qualitative assessment of the performance, a ground truth map inspired by biological images of vascular networks was prepared [Fig. 3(b)]. This included a radial network of vessels with decreasing vessel diameter at the periphery of the image. Inside these vessels the total hemoglobin abundance was set to a value of 1. Outside vessels, the total hemoglobin abundance was set to a value of 0.5 to represent regions where the signal contribution from small unresolvable capillaries is mixed with the non-vascular background, ultimately representing a homogenous tissue background.

## 3. Results

**Fig. 4.**
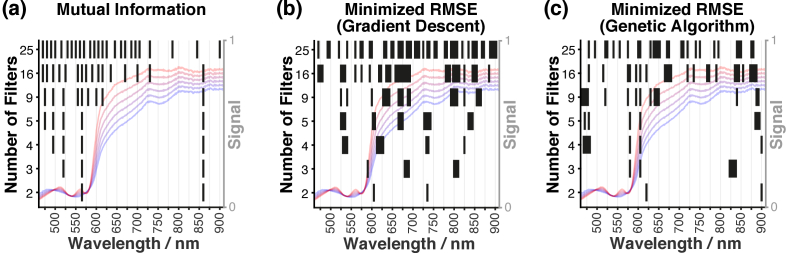
**The selected filters for *n* = 2, 3, 4, 5, 9, 16 and 25.** Filters are shown as black bars with the center wavelength represented by the center position of the bar and the FWHM represented by the width of the bar. **(a)**. Filters optimized by minimizing mutual information. **(b).** Filters optimized by minimizing RMSE via gradient descent. **(c).** Filters optimized by a minimizing RMSE via genetic algorithm. Example signal hypercube spectra for *A_THb_* = 1 and *SO_2_ *= 0, 0.2, 0.4, 0.6, 0.8 and 1.0 are shown in faint blue to red respectively to allow comparison with the selected filter sets.

Spectral filter sets were chosen using four methods: evenly spaced filters, like those found in an ‘off-the-shelf’ system (1); filters optimized by minimizing mutual information between filters (2); and filters optimized by minimizing the RMSE in hemoglobin abundance prediction, using gradient descent (3); and using a genetic algorithm (4). The selected filters for *n* = 2, 3, 4, 5, 9, 16 and 25 are shown in Fig. 4 for mutual information, gradient descent, and genetic algorithms. The time taken for each method is shown in Table S1. The selected filters for all *n* are shown in Fig. S2. The hierarchical clustering of filters selected by minimizing mutual information is evident in Fig. 4(a). A heat map of mutual information is shown in Fig. S3. The filters optimized by gradient descent [Fig. 4(a)] have center wavelengths close to the evenly spaced starting center wavelengths, suggesting the algorithm became trapped in local minima. In contrast, the filters optimized by genetic algorithm [Fig. 4(c)] are more unevenly distributed suggesting a better global optimization.

**Fig. 5.**
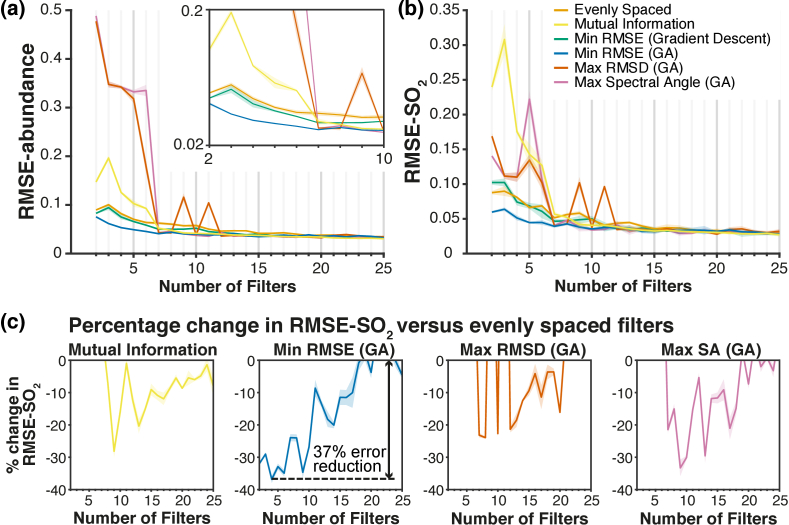
**Filter set performance versus number of filters for each optimization method. (a)** The root-mean-square-error (RMSE) in estimated abundance of HbO_2_ and Hb as calculated by Eq. (22). Shaded region is 5 × standard deviation of results from 3 test images. **(b)** The RMSE in the estimated SO_2_ (RMSE-SO_2_) for *A_THb_^true^ *= 0.8–1.0. Shaded region is 5 × standard deviation of results from 3 test images. **(c)** The percentage change in RMSE-SO_2_ versus evenly spaced filters for *A_THb_^true^ *= 0.8–1.0. GA, genetic algorithm; RMSD, root-mean-square-difference; SA, spectral angle. Shaded region is the standard deviation of results from 3 test images.

The performance of each filter set was determined by simulating imaging of an independent test hypercube generated using the gradient abundance map shown in Fig. 3(a). Following spectral unmixing, the root-mean-square-error (RMSE) in estimated abundance **[Fig. 5(a)]** and SO_2_ [Fig. 5(b)] were calculated. For high numbers of filters, *n *> 15, there is little absolute difference in performance between filter sets selected using each of the four methods, as most of the wavelength range is sampled. However, for sparse sampling (small numbers of filters; *n *< 10), filter sets optimized using a genetic algorithm result in superior performance to filters optimized by all other approaches, with up to a 37% reduction in RMSE-SO_2_ compared to evenly spaced filters [Fig. 5(c)]. For example, optimization of *n *= 3 filters using the genetic algorithm results in a 39% decrease in RMSE-abundance (0.06 vs. 0.10) and a 29% decrease in RMSE-SO_2_ (0.064 vs. 0.090) compared with evenly spaced filters.

Optimization based on the alternative merit functions (Section 2.7) that maximize spectral angle (SA) and root-mean-square-difference (RMSD) between HbO_2_ and Hb spectra perform poorly at low *n*, but better at *n *> 7, as do filters optimized using mutual information, as can be seen in the percentage reduction of RMSE-SO_2_ versus evenly spaced filters [Fig. 5(c)], but at high filter numbers, this absolute improvement is small [Fig. 5(b)].

**Fig. 6.**
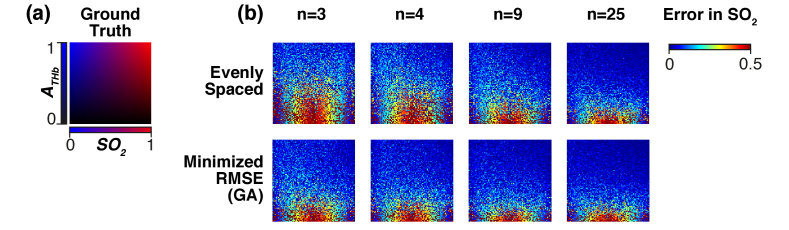
**Error in estimated oxygen saturation. (a)** The ground truth abundance. The red channel represents the abundance of oxyhemoglobin. The blue channel represents the abundance of deoxyhemoglobin. Thus, the color value from black–color represents ground truth total hemoglobin abundance *A_THb_* = 0–1. **(b)** The absolute error in estimated SO_2_ for imaging with n = 3, 4, 9 and 25 evenly spaced filters and filters optimized by minimizing root-mean-square-error (RMSE) via genetic algorithm (GA).

The error in estimated SO_2_ is shown in Fig. 6 for evenly spaced filter sets and optimized filter sets for *n* = 3, 4, 9 and 25. In all cases the error in estimated SO_2_ is largest at low THb abundance due to lower signal to noise ratio. For *n* = 3 and *n* = 4, the error in SO_2_ for intermediate *A_THb_*∼0.3–0.6 is significantly lower using optimized filter sets than using evenly spaced filter sets.

**Fig. 7.**
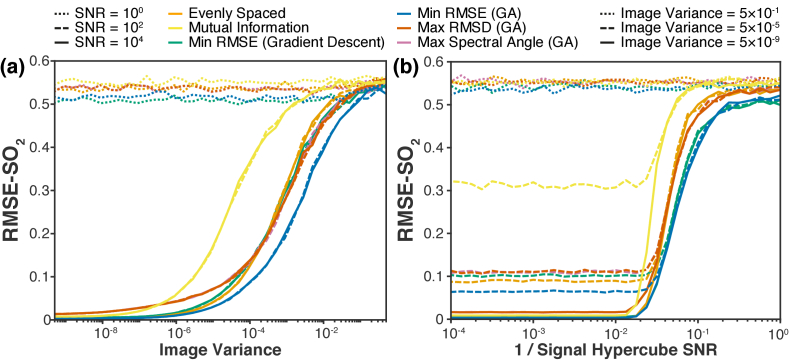
**The effect of noise on performance. (a)** Root-mean-square-error in estimated SO_2_ (RMSE-SO_2_) versus test image noise variance for signal hypercubes with SNR = 10^0^, 10^2^ and 10^4^. **(b)** Root-mean-square-error in estimated SO_2_ (RMSE-SO_2_) versus 1 / signal hypercube SNR for test image noise variance = 5 × 10^−1^, 5 × 10^−5^ and 5 × 10^−9^. *A_THb_^true^ *= 0.8–1.0.

To assess the effects of noise, gradient test images [Fig. 3(a)] with various degrees of gaussian white noise added to the signal hypercube and various degrees of gaussian white noise added to the simulated images were generated. These images were ‘imaged’ with the optimized filter sets for *n = *3, unmixed, and root-mean-square-error in estimated SO_2_ (RMSE-SO_2_) calculated. The results are shown in Fig. 7. Filter sets optimized to minimize RMSE via genetic algorithm remained the highest performing filter sets across noise conditions.

**Fig. 8.**
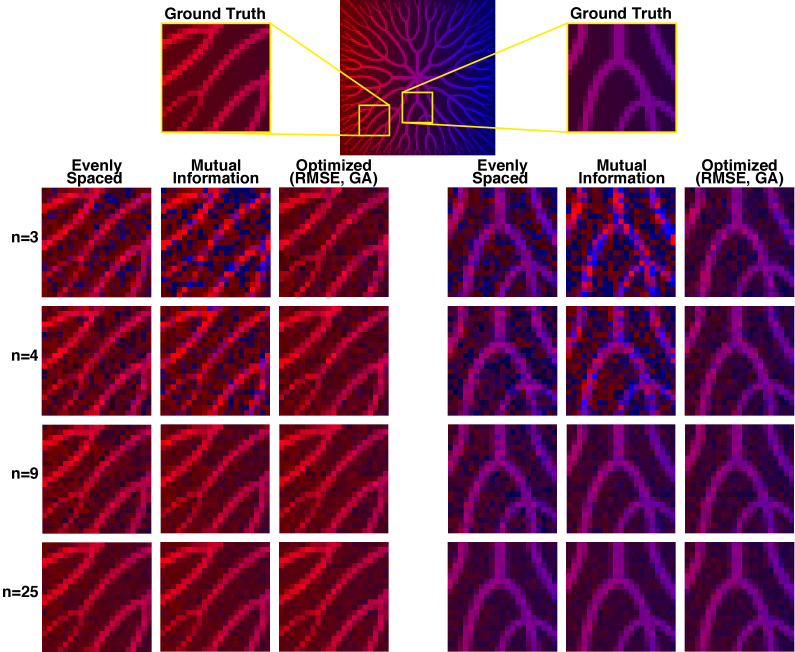
**Example filter set performance for imaging of biologically inspired vessels.** The ground truth abundance maps for two regions of the vessel image are shown (top). For each of these regions, the estimated abundance maps are shown for imaging with *n* = 3, 4, 9 and 25 filters selected using three different methods: evenly spaced filters, filters optimized by minimizing mutual information and filters optimized by minimizing root-mean-square-error (RMSE) via a genetic algorithm (GA). The red channel represents the abundance of oxyhemoglobin. The blue channel represents the abundance of deoxyhemoglobin.

Figure 8 shows the estimated abundance maps for imaging with *n* = 3, 4, 9 and 25 filter sets selected using three different methods: evenly spaced filters, filters minimizing mutual information and filters optimized using a genetic algorithm (GA). Further examples are shown in Fig. S4. For *n* = 16 and *n* = 25, images are similarly accurate for filter sets chosen by all methods. For *n* = 3 and *n* = 4, the images simulated using optimized filters are visibly less noisy and more accurate than the images simulated using evenly spaced filters and filters selected to minimize mutual information. Indeed, the latter are particularly inaccurate, showing many pixels with low SO_2_ (blue) where ground truth SO_2_ is high.

## 4. Discussion

The results clearly demonstrate the merit of tailoring spectral filter sets towards specific biomedical problems. The RMSE in determining SO_2_ was 24–37% lower when imaging with *n *= 2–10 optimized spectral filters compared with the same numbers of evenly spaced filters. Moreover, the results make clear the importance of performing this optimization with the end-goal clinical challenge in mind by using appropriate functions of merit to assess filter set performance, in this case the RMSE in estimating hemoglobin abundances. Filter sets optimized using a merit function based on accurate estimation of hemoglobin abundance resulted in significantly more accurate estimation of SO_2_ compared with spectral filter sets chosen to minimize mutual information, maximize spectral angle between HbO_2_ and Hb spectra or maximize root-mean-square-difference between HbO_2_ and Hb spectra (69–79%, 43–80% and 43–67% decrease in RMSE-SO_2_ for imaging with *n = *2–5 filters chosen to minimize mutual information, maximize spectral angle between HbO_2_ and Hb spectra or maximize root-mean-square-difference between HbO_2_ and Hb spectra respectively).

The results also show that optimized filter sets consistently perform better than non-optimized or poorly optimized filter sets across a wide range of noise levels. The noise levels in experimental images will depend on the experimental setting as well as the acquisition parameters such as exposure time and illumination power. In standard practice, we can assume these would be adjusted by the user to achieve adequately small noise levels and ensure an optimized filter set’s performance matches that seen in simulation. Otherwise, we suggest users perform their own optimization using noise levels tuned to those expected in their own experimental setups.

Now is the time to perform spectral filter optimizations. Many spectral imaging approaches are on the cusp of clinical translation, but despite the number of publications, very few *in vivo* studies have been conducted [27]. Commercial devices for hyperspectral imaging of oxygenation, such as the TIVITA series imagers (Diaspective Vision GmbH, Germany), HyperView (Hypermed Imaging, Inc., USA) and Snapshot NIR (Kent Imaging, Inc., Canada), are rapidly emerging, and multiple hyperspectral imaging start-ups have been founded in the past few years. It is our belief that wider adoption of proper spectral band selection will result in more effective multispectral imaging systems and consequently, more widespread adoption of multispectral imaging technologies in clinic.

We foresee a future where ubiquitous ‘one-size-fits-all’ RGB imaging systems in endoscopy, laparoscopy and surgical microscopy are replaced with application-specific multispectral imaging systems tailored to the measurement of biological signatures relevant to the said application. Proponents of the ‘one-size-fits-all’ approach might point out the increased cost associated with purchasing and maintaining multiple application-specific imaging tools, but this is already the norm in surgical instruments, where a plethora of application-specific scalpels, forceps, scissors, retractors, and clamps have long been available. Still, the relatively high cost of imaging sensors might previously have disqualified this comparison, but recent innovations in optical filter fabrication [42,43] increasingly allow for low-cost manufacture of custom sensors.

An alternative and promising approach to multispectral imaging is the use of sequential spectral filtering of the illumination, allowing the use of common monochrome detectors, as is already in use in flexible endoscopy for RGB, autofluorescence imaging and NBI. The increasing availability of high-power narrow-band LEDs might facilitate the use of multi-LED arrays for illumination. This would enable the continued use of ubiquitous ‘one-size-fits-all’ hardware, with software instructing the multi-LED array to provide application-specific sequential illumination as determined by spectral filter optimization per indication.

We have identified several limitations to this work. To ensure applicability across acquisition approaches, the present study did not consider the trade-off between increasing the number of filters and decreasing spatial and/or temporal resolution, but this has been explored previously for multispectral filter arrays. Ultimately, the trade-off between spectral and temporal resolution will be a somewhat subjective choice that depends on the intended end-users and application. Another limitation of the current study is the use of standard fitting by minimization of a sum of square errors cost function to fit the empirical model to the simulated images and determine estimated abundances. Increasingly, convolutional neural networks (CNNs) are being used to improve the interpretation of biomedical images. An embedded CNN-based approach to spectral band selection should be explored for use in biomedical spectral band optimization once appropriate labelled datasets are available [44]. Finally, the present study used a simple empirical model of reflectance; future work may expand this to a 3D tissue model using Monte Carlo photon transport simulations. Nevertheless, the empirical model’s good fit to experimental esophageal tissue data is encouraging. For future work, we advise users to employ experimentally acquired ground truth hypercubes as inputs, so optimization is based on the most accurate spectral properties of the tissue of interest.

In the present study we chose to focus on Gaussian filter profiles as these can be used to approximate the spectral filter profiles of narrow-band light sources (e.g. LED arrays), tunable filters (e.g. LCTFs, AOTFs) and spectrally resolved detector arrays (e.g. micro-pixelated filters). Still, other users may require optimization of different filter shapes (e.g. dichroic band pass). The presented algorithm is designed such that alternative filter shapes could be added as inputs to the optimization, and we encourage users to perform optimizations to suit their needs. The results of the present study demonstrate the potential of spectral band optimization, and we hope this will persuade researchers to use the presented algorithms, with their own inputs, to enable optimization tailored towards their own specific applications.

## 5. Conclusions

We demonstrate the optimization of spectral filter sets enables more accurate estimation of oxygen saturation in spectral imaging, with up to a 37% reduction in root-mean-square-error compared with the use of generalist sensors. This work clearly demonstrates the merit of tailoring spectral filter sets towards specific biomedical problems. Wider adoption of this approach will result in more effective multispectral image systems and consequently, more widespread adoption of multispectral imaging technologies in clinic.

## Data Availability

Data underlying the results presented in this paper are available in Ref. [45].

## Supplemental document

See Supplement 1 for supporting content.
