# Supplementary material for: Optimized spectral filter design enables more accurate estimation of oxygen saturation in spectral imaging
Source: Biomed Opt Express. 2022 Mar 16;13(4):2156–73. doi: 10.1364/BOE.446975 (PMC9045927; doi:10.1364/BOE.446975)
Supplement: Supplementary file 1 [file boe-13-4-2156-s001.pdf]

Supplemental Document

Biomedical Optics EXPRESS

Optimized spectral filter design enables more accurate estimation of oxygen saturation in spectral imaging: supplement

DALE J. WATERHOUSE[1,2] AND DAN STOYANOV[1,3]

[1] *Wellcome/EPSRC Centre for Interventional and Surgical Sciences, Department of Medical Physics and Biomedical Engineering, University College London, UK*
[2] *d.waterhouse@ucl.ac.uk*
[3] *danail.stoyanov@ucl.ac.uk*

This supplement published with Optica Publishing Group on 16 March 2022 by The Authors under the terms of the Creative Commons Attribution 4.0 License in the format provided by the authors and unedited. Further distribution of this work must maintain attribution to the author(s) and the published article's title, journal citation, and DOI.

Supplement DOI: https://doi.org/10.6084/m9.figshare.19121669

Parent Article DOI: https://doi.org/10.1364/BOE.446975

Optimized spectral filter design enables more accurate estimation of oxygen saturation in spectral imaging: supplemental document

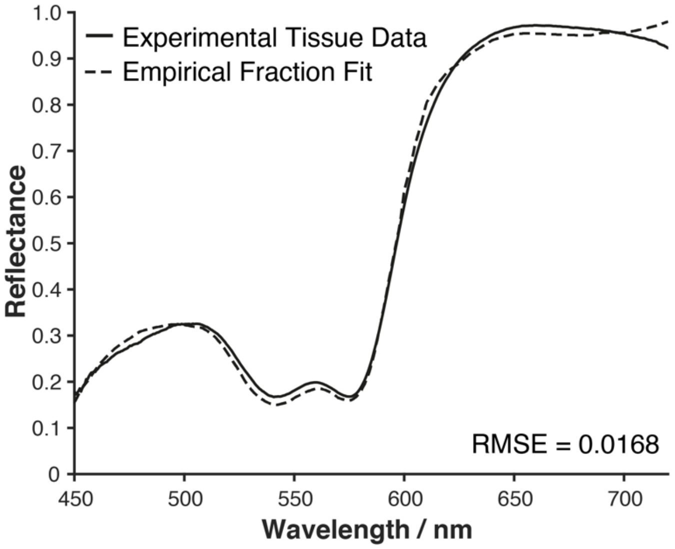

Fig. S1. Empirical model of tissue reflectance fitted to experimental diffuse reflectance from esophageal tissue. An empirical model of tissue reflectance, $R(\lambda) = \mu'_s(\lambda) / (k_1\mu_a(\lambda) + k_2)$, was fitted to the mean of 320 diffuse reflectance spectra captured from *in vivo* human esophageal tissue in a previous study [1]. Databook values of μ'_s and μ_a were taken from [2]. This determined the values of k_1=0.26 and k_2=14. The root mean square error (RMSE) of the fit was 0.0168.

Method	Time taken to optimize n filters / seconds			
	n=3	n=9	n=16	n=25
Evenly Spaced	~0	~0	~0	~0
Mutual Information	0.63	0.60	0.61	0.64
Min RMSE (Gradient Descent)	190	2400	4700	3100
Min RMSE (GA)	8400	6900	3700	1600
Max RMSD (GA)	48	130	280	28
Min Spectral Angle (GA)	32	152	127	29

Table S1. Computation times for each of the optimization methods for *n* = 3, 9, 16 and 25 filters.

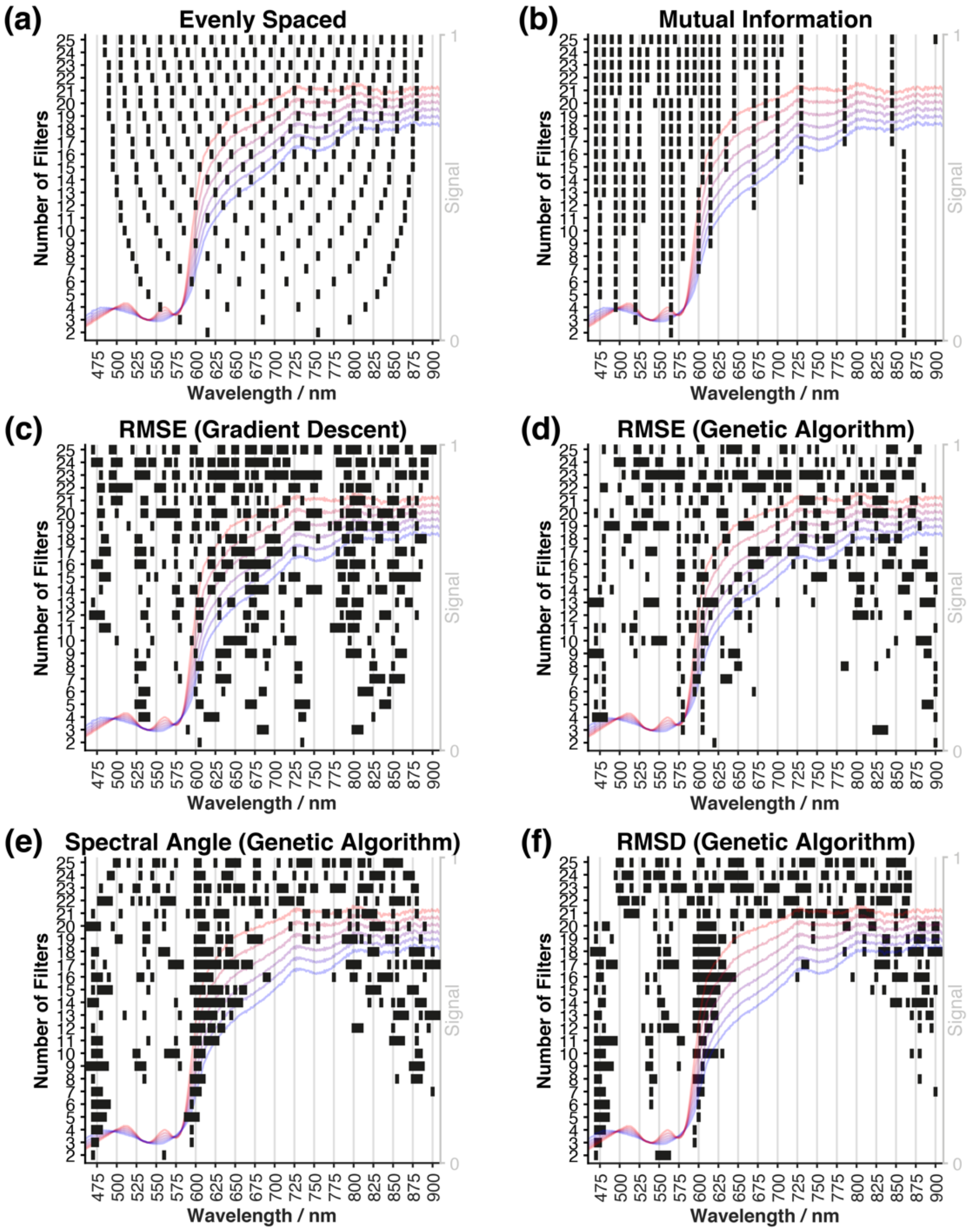

Fig. S2. The selected filters for n = 2–25. Filters are shown as black bars with the center wavelength represented by the center position of the bar and the FWHM represented by the width of the bar. **(a)**. Evenly spaced filters. **(b).** Filters optimized by minimizing mutual information. **(c).** Filters optimized by minimizing RMSE via gradient descent. **(d).** Filters optimized by minimizing root-mean-square-error (RMSE) via genetic algorithm. **(e)** Filters optimized by maximizing spectral angle via genetic algorithm. **(f)** Filters optimized by maximizing root-mean-square-difference (RMSD) via genetic algorithm. Example ground truth spectra for A_{THb} = 1 and SO_2 = 0, 0.2, 0.4, 0.6, 0.8 and 1.0 are shown in faint blue to red respectively to allow comparison with the selected filter sets.

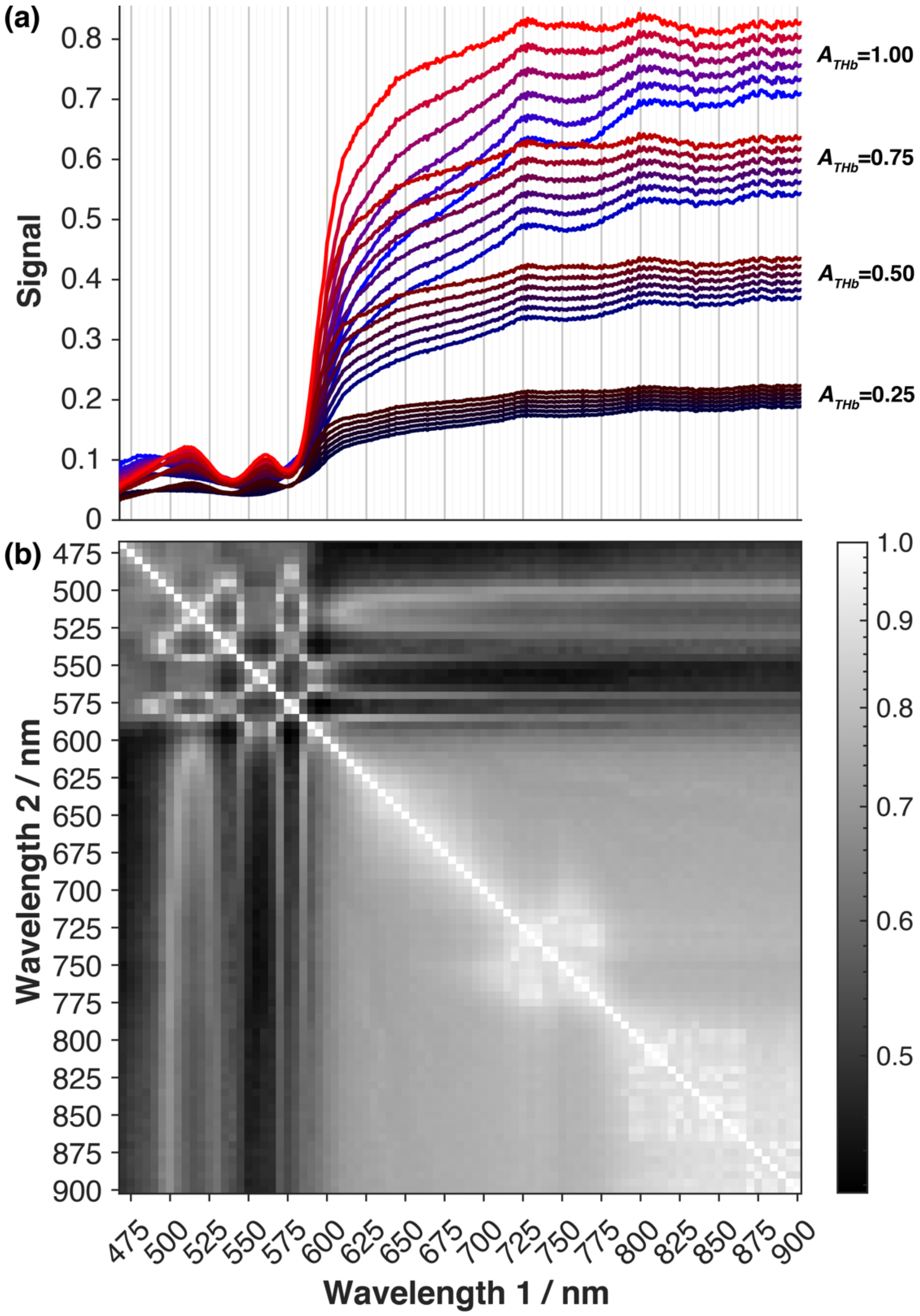

Fig. S3. Mutual Information. (a). Example spectra from the model hypercube with total abundance A_{THb} = 0.25, 0.5, 0.75 and 1 and SO_2 = 0, 0.2, 0.4, 0.6, 0.8 and 1. **(b).** Normalized mutual information in the ground truth signal hypercube (see Eq. 32 in the main manuscript).

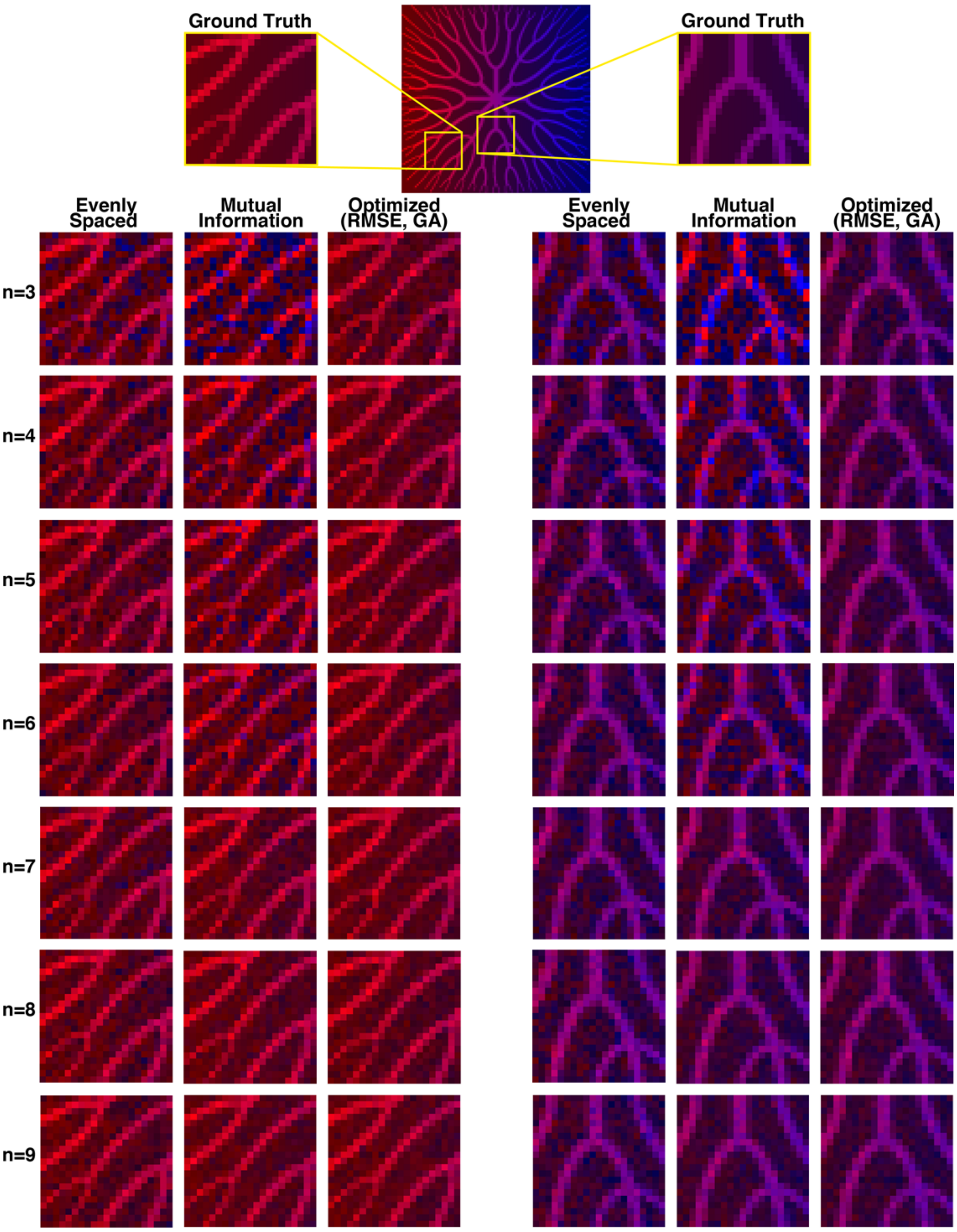

Fig. S4. Example filter set performance for imaging of biologically inspired vessels. The ground truth abundance maps for two regions of the vessel image are shown (top). For each of these regions, the estimated abundance maps are shown for imaging with n = 2, 3, 4, 5, 6, 7, 8 and 9 filters selected using three different methods: evenly spaced filters, filters optimized by minimizing mutual information and filters optimized by minimizing root-mean-square-error (RMSE) via a genetic algorithm (GA). The red channel represents the abundance of oxyhemoglobin. The blue channel represents the abundance of deoxyhemoglobin.

References

1. D. J. Waterhouse, W. Januszewicz, S. Ali, R. C. Fitzgerald, di pietro Massimiliano, and S. E. Bohndiek, "Spectral endoscopy enhances contrast for neoplasia in surveillance of Barrett’s esophagus," Cancer Research **81**, 3415–3425 (2021).
2. N. Bosschaart, G. J. Edelman, M. C. G. Aalders, T. G. van Leeuwen, and D. J. Faber, "A literature review and novel theoretical approach on the optical properties of whole blood," Lasers in Medical Science **29**(2), 453–479 (2014).
